# Transition of Thai HIV-infected adolescents to adult HIV care

**DOI:** 10.7448/IAS.18.1.20651

**Published:** 2015-12-03

**Authors:** Rawiwan Hansudewechakul, Supawadee Pongprapass, Areerat Kongphonoi, Sukanda Denjanta, Suporn Watanaporn, Annette H Sohn

**Affiliations:** 1 Department of Pediatrics, Chiangrai Prachanukroh Hospital, Chiang Rai, Thailand; 2 Department of Medicine, Chiangrai Prachanukroh Hospital, Chiang Rai, Thailand; 3 TREAT Asia/amfAR, The Foundation for AIDS Research, Bangkok, Thailand

Global successes in increasing the numbers of people living with HIV on antiretroviral therapy (ART) have turned HIV into a chronic disease. However, disease control is entirely dependent on lifelong adherence to ART and retention in clinical care. This is a particular challenge for perinatally infected children and adolescents, who may not remember a time in their lives when they did not have to take medicines. When these children become young adults, many find themselves having to transition their care from paediatric to adult HIV providers. There are few reports of what is actually happening around the time of transition and whether they successfully remain in care. More transition models and tracking of clinical outcomes are needed to inform local standards of care. The following is one example reflecting seven years of experience in northern Thailand.

Chiangrai Prachanukroh Hospital (CRH) is a 750-bed public hospital located in Chiang Rai province, Thailand. The paediatric outpatient department of CRH has started ART in 633 children and adolescents since 2003. The majority of the patients who have passed through our clinic have been referred back to one of 16 community hospitals that are members of the Chiang Rai Children and Adolescent ART Network. Of the 216 children and adolescents who remain in active follow-up at the CRH paediatric clinic, 192 are over 10 years of age and 130 are over 15 years of age. With each passing year, there are more patients who reach young adulthood. Through March 2015, 67 adolescents who had aged out of the paediatric clinic had been directly transferred to the CRH adult outpatient HIV clinic.

In order to manage the numbers of patients who needed to be transferred to adult care, in 2008 the paediatric department developed a group transition programme that prepares cohorts of adolescents, rather than focusing on individual patients. Paediatric providers initially identify adolescents who are transition candidates. All have already had their HIV status disclosed to them through a process that the clinic usually initiates at the age of 7 to 10 years. The paediatric HIV clinic team then organizes multiple meetings to review the list of transition candidates and plan for the next round of preparatory activities. After these case conferences, the patients’ medical records are prepared for the adult care providers, who then orient themselves to the patients’ clinical histories. To improve the efficiency and acceptability of the transition process for providers at CRH, interdepartmental case conferences are arranged before and after each group transition event for paediatric providers, adult care providers, the hospital's home care team and clinical psychology consultants.

The transitioning adolescents are invited to participate in a transition camp that focuses on antiretroviral management, HIV transmission and learning how to navigate the adult HIV care setting. The camps take place over one to two days at an off-site location, typically in a rural area outside of the city. During the camps, the adolescents are given the opportunity to meet and speak with adult HIV care providers and to speak with each other about how they will manage their own HIV care in the future. After the camp, the youth are taken with their peers to the adult HIV department at CRH and shown where they will go for different services. All of the youth who participated in the camp together will be scheduled to have the same initial appointment day and time in the adult clinic. This arrangement allows the group to go to the hospital for their individual clinic visits, sit in the waiting area, get their blood drawn, attend a counselling session and pick up their medicines together. A paediatric provider is also present at the few first adult medical appointments for each transition group to provide immediate support and to answer questions. There have been cases where adolescents were ready to transition as individuals, but in numbers too small to support a group transition event. These patients are offered similar transition preparation, but without group support.

The first transition group in 2008 included 17 adolescents, and the second group of 22 transitioned in 2009. The 2010 transition camp was delayed because the patients felt that they needed more time to prepare and were not ready to leave the paediatric clinic. The paediatric HIV care team then decided to delay transition until the age of at least 18 years. In 2013, 10 youth moved to adult HIV care. In addition, 18 individual youths were transitioned outside of the group process between 2010 and 2014.

## Adolescent-to-adult transition outcomes

The first and second groups are now more than five years post-transition. The third group is two years post-transition. Of the 49 (73%) who remain in active follow-up at CRH, half are on second-line protease inhibitor-based ART regimens, and 37 (76%) were virologically suppressed in the previous year (Table 1). The four deaths took place both soon after (2.5 and 7 months) and long after (2.6 and 3.9 years) transition. All of these patients had unsuppressed virus at the time of transition (4500 to 149,000 copies/ml) and challenges with poor adherence.

**Table 1:** Transitioned HIV-infected adolescents, Chiangrai Prachanukroh Hospital, March 2015

Group and transition year	Group 1 2008 *n* (%)	Group 2 2009 *n* (%)	Group 3 2013 *n* (%)	Individuals 2010 to 2014 *n* (%)	Total *n* (%)
Post-transition duration	>6 years	>5 years	2 years	1 to 5 years	
Number of adolescents	17	22	10	18	67
Deaths	1 (6)	2 (9)	0	1 (6)	4 (6)
Referral to outside hospitals	1 (6)	2 (9)	1 (10)	1 (6)	5 (7)
Lost to follow-up	3 (18)	2 (9)	0	4 (22)	9 (13)
Active follow-up	12 (71)	16 (73)	9 (90)	12 (67)	49 (73)
NNRTI-based ART^a^	4 (33)	9 (56)	8 (89)	5 (42)	26 (53)
PI-based ART^a^	8 (67)	7 (44)	1 (11)	7 (58)	23 (47)
Current VL, copies/ml^a^					
<40	11 (92)	10 (63)	9 (100)	7 (58)	37 (76)
>40 to 1999	0	3 (19)	0	0	3 (6)
>2000	1 (8)	3 (19)	0	5 (42)	9 (18)

a Regimen and viral load data available only for patients in active follow-up at the hospital's adult HIV clinic. Total in active follow-up used as the denominator to calculate percentages; NNRTI, non-nucleoside reverse transcriptase inhibitor; ART: antiretroviral therapy; PI: protease inhibitor; VL: viral load.

Our experience has been that transitioning youth in groups and having them go to the adult HIV department together creates a support system for the process. This arrangement also facilitates positive peer interactions, where virologically suppressed adolescents are able to support others who are struggling with individual issues, such as poor medication adherence, alcohol use, difficult family relationships or other factors that may be preventing them from transitioning. Although our transition outcomes were observed in the setting of a provincial-level referral hospital, we have used our experiences in managing disclosure and transition to help us provide ongoing technical support to a local network of 16 community hospitals. Our programme has demonstrated that adolescents can be successfully transitioned in the context of a supportive process that involves both paediatric and adult healthcare providers and respects the individual patient's readiness to transfer care.

